# Single-dose versus multi-dose steroid therapy in pediatric nephrotic syndrome: a systematic review and meta-analysis

**DOI:** 10.1007/s00467-026-07175-z

**Published:** 2026-02-28

**Authors:** Candela Romano, Lisyareli Salazar Arriaga, Linda Estefania Cordero Velez, Alexandra Melendez Delgado, Tatiana Alexandra Moreno Díaz, Haya Al Shakkakee, Abhigna Reddy Kudumula, Steysi Connie Falcon Aragon, Ernesto Calderon Martinez

**Affiliations:** 1 University of California, 101 The City Drive South, ZC4482, Orange, Irvine, CA 92868 USA; 2 Universidad de Monterrey, Monterrey, México; 3 Universidad del Azuay, Cuenca, Ecuador; 4 Universidad del Rosario, Bogota, Colombia; 5 Facultad de Salud, Universidad Industrial de Santander, Bucaramanga, Colombia; 6 University of Baghdad/Al Kindy College of Medicine, Baghdad, Iraq; 7 Mallareddy Medical College for Women, Hyderabad, India; 8 Universidad Ricardo Palma, Lima, Peru; 9 University of Texas at Houston Medical Center, Houston, TX USA

**Keywords:** Adverse events, Corticosteroids, Nephrotic syndrome, Pediatrics, Steroids, Divided-dose, Single-dose, Recurrences, Relapse, Remission

## Abstract

**Background:**

Corticosteroids remain the cornerstone of therapy for steroid-sensitive nephrotic syndrome (SSNS), yet the optimal dosing strategy, single daily versus divided doses, remains uncertain.

**Methods:**

We conducted a systematic review of studies comparing once-daily and divided-dose prednisolone in children with SSNS. Three randomized controlled trials and one non-randomized study were included, enrolling a total of 288 patients. Primary outcomes were time to remission and relapse frequency; secondary outcomes included hypothalamic–pituitary–adrenal (HPA) axis suppression and adverse events.

**Results:**

Overall, remission rates were comparable between single and divided dosing. In relapse settings, one trial showed faster remission with split dosing (by 1–2 days), though without differences in relapse frequency or safety. In first-episode disease, single daily dosing was associated with less HPA axis suppression and a longer time to first relapse. Adverse event profiles were similar across regimens.

**Conclusions:**

Single-dose and split-dose prednisolone regimens demonstrate comparable remission efficacy in children with SSNS. Divided dosing may shorten remission by 1–2 days during relapse episodes, but this small benefit must be weighed against the greater HPA-axis suppression observed with multi-dose schedules. Given the heterogeneity and small sample sizes of available trials, the current evidence remains insufficient to establish superiority. Larger multicenter RCTs with standardized protocols and longer follow-up are needed to provide more definitive guidance.

**Graphical abstract:**

A higher resolution version of the Graphical abstract is available as Supplementary information

**Figure:**
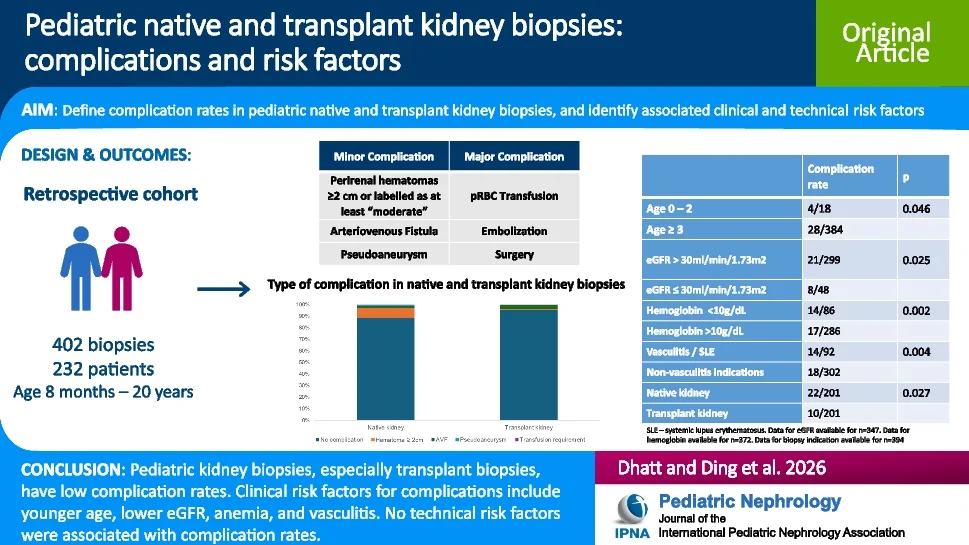

**Supplementary Information:**

The online version contains supplementary material available at https://doi.org/10.1007/s00467-026-07175-z.

## Introduction

Nephrotic syndrome (NS) is the most common glomerular disease in children, with an annual incidence ranging from 1.2 to 16.9 per 100,000 worldwide [1]. Its pathophysiology remains incompletely understood, though it is believed to result from a combination of immune dysregulation and, in some cases, genetic mutations [2]. Approximately 85–90% of pediatric cases present as steroid-sensitive nephrotic syndrome (SSNS), which is typically characterized by edema, hypoalbuminemia, and heavy proteinuria [1, 3].

International guidelines, including those from the International Pediatric Nephrology Association (IPNA) and Kidney Disease: Improving Global Outcomes (KDIGO), recommend an initial 8–12-week course of oral glucocorticoids for children with new-onset SSNS, typically prednisone or prednisolone at 60 mg/m^2^/day for 4–6 weeks, followed by 40 mg/m^2^ on alternate days [3, 4]. However, these guidelines do not specify whether steroids should be given as a single morning dose or divided into multiple daily doses. As a result, uncertainty remains about whether dosing schedules influence remission rates, relapse frequency, or the risk of steroid-related toxicity [4, 5].

Emerging evidence suggests that once-daily dosing may reduce hypothalamic–pituitary–adrenal (HPA) axis suppression and prolong the time to first relapse, while achieving remission rates comparable to divided dosing [6]. Conversely, some studies indicate that split dosing may accelerate remission during relapses, though without consistent differences in long-term outcomes or adverse effects [7]. Observational data further highlight that once-daily regimens may be easier for families to follow, potentially improving adherence and simplifying care [8].

Optimizing glucocorticoid dosing in pediatric NS is critical to balance efficacy with safety. For children, selecting the most effective, least toxic, and most practical regimen has important implications for long-term health outcomes and quality of life.

Endogenous cortisol secretion exhibits a strong circadian pattern with an early-morning peak. Administering prednisolone as a once-daily morning dose aligns exogenous exposure with this physiology and may mitigate HPA-axis suppression relative to schedules extending into the evening ​[6]​. From a practical standpoint, once-daily regimens can also enhance adherence and reduce treatment burden for families ​[8]​. In contrast, divided dosing may sustain glucocorticoid receptor occupancy across the day and has been associated with slightly faster remission during relapse episodes ​[18, 19]​. On this basis, we evaluated whether once daily and divided dosing differ in efficacy (time to remission, relapse) and endocrine safety (HPA suppression), while keeping the total daily dose equivalent.

This systematic review evaluates single versus divided steroid dosing in pediatric NS, focusing on remission rates, relapse frequency, endocrine safety, and adherence. The aim is to provide evidence that can guide clinical practice and inform future guideline updates.

## Methods

The present systematic review followed the recommendations and criteria established by the Preferred Reporting Items for Systematic Reviews and Meta-analyses (PRISMA) reporting guidelines [9]. The protocol was pre-registered at the International Prospective Register of Systematic Review (PROSPERO) with the identifier code (CRD420251088493).

## Eligibility criteria

### Types of participants

The inclusion criteria consist of children under 18 years old with NS, defined as patients with gross proteinuria (+ +  + or more) with UPcr > 200 mg/mmol, hypoalbuminemia (albumin < 2.5 g/dl), and edema. Exclusion criteria include participants older than 18 years old.

### Types of interventions

Eligible interventions included once-daily steroid regimens. The comparison group consisted of patients who received divided (multiple daily) steroid doses. Only studies that directly compared single-dose versus multiple-dose steroid regimens were included.

### Outcomes

Studies were included if they reported at least one of our predefined outcomes of interest. Our primary efficacy outcomes were: (1) time to remission, measured in days from the start of prednisolone therapy, and (2) relapse-related outcomes, including relapse frequency during follow-up, whether a relapse occurred, and, when available, the time to first relapse. Secondary outcomes included adverse events and HPA-axis suppression. Adverse events encompassed infections, Cushingoid features, behavioral changes, gastrointestinal symptoms, serious infections, obesity, and skin infections such as impetigo, as described by each individual trial. Objective assessment of HPA-axis suppression was available in only one study. Although growth parameters (including height) were part of our prespecified outcomes, none of the included studies reported longitudinal growth data, and therefore, these outcomes could not be evaluated.

### Types of studies

This systematic review and meta-analysis includes studies published from the inception of the database, restricted to those available in English and Spanish. Eligible study designs include randomized clinical trials (RCTs), crossover studies, and non-randomized comparatives studies. Excluded studies consist of reviews, protocol articles, books, conference abstracts, editorials, letters to editor, news articles, or other non-original research. Additionally, studies were excluded if they are duplicates, lack methodological detail, or if the necessary data could not be obtained after attempts to contact the original authors.

### Search methods

A systematic search was initially conducted on July 9, 2025, on PubMed MEDLINE, Cochrane, Scopus, Web of Science, EMBASE, CINAHL, and Google Scholar using the following terms: “nephrotic syndrome”, “pediatrics”, “single dose steroid”, “multiple-dose steroid”.

### Selection of studies

All references were exported to Rayyan (Rayyan Systems Inc., Cambridge, MA, USA), and duplicates were removed [10]. Two authors independently completed the eligibility assessment, first by title and abstract analysis and, subsequently, by full-text assessment. In disagreements between reviewers, a third reviewer helped reach a consensus.

### Assessment of risk of bias in included studies

The methodological quality of the included studies was assessed using the Cochrane Risk of Bias 2.0 (RoB 2.0) tool for randomized controlled trials and the ROBINS-I tool for the non-randomized studies. Each study was evaluated across five domains: randomization process, deviations from intended interventions, missing outcome data, measurement of outcomes, and selective reporting. Risk of bias was categorized as low, some concerns, or high. Two independent reviewers evaluated the risk of bias in each study, considering the specific criteria and guidelines provided by the respective tools. Any reviewer discrepancies were resolved through discussion with a third blinded reviewer [11, 12].

### Data extraction

Two independent reviewers extracted the data; disagreements were resolved by consensus. When multiple overlapping reports from the same study were identified, the information from the one containing the most relevant information or the first published report was included. Extracted data included first author, publication year, country, study design, participants’ age, sample sizes, intervention types (single vs. multiple dose), and measured outcomes. Conventional methods were used for data extraction, complemented by specialized tools such as WebPlotDigitizer (Automeris, Austin, TX, USA) for digitizing data from graphs, Cochrane Calculator for statistical conversions, and StatsToDo for advanced calculations. Additional extracted data included subgroup characteristics, such as country of study, risk of bias levels, intervention dose, and frequency [13–15].

### Statistical analysis

A meta-analysis was performed using R version 3.4.3 (R Core Team) [16]. The pooled effect of the outcomes was examined using a random-effects meta-analysis (DerSimonian-Laird approach) [17]. Whenever the number of studies reporting an outcome of interest was insufficient, only a qualitative analysis of the results was performed. Effect sizes were expressed as relative risk (RR) or standardized mean difference (SMD) with a 95% confidence interval. The I2 statistic assessed heterogeneity, and the following cut-off values were used for interpretation: < 25, 25–50, and > 50% were considered small, medium, and large heterogeneity, respectively. For all outcomes, sensitivity analyses using the leave-one-out method were performed to determine the influence of individual studies on the overall effect. Egger's regression test was used to examine publication bias when 10 or more reports with the same outcomes were available. Whenever possible, subgroup analyses were performed for primary outcomes. Because only four eligible trials were identified, separate quantitative analyses for first episode versus relapse settings were not feasible. Therefore, we pooled all trials using time to remission as a common outcome while acknowledging the clinical heterogeneity this approach introduces.

## Results

A total of 2,927 records were identified from registers and six databases, including PubMed (n = 315), Cochrane (n = 616), Scopus (n = 280), Web of Science (n = 1,125), CINAHL (n = 51), and Embase (n = 540). After removing 1,449 duplicates, 1,478 records remained for screening. Following title and abstract screening, 1,455 records were excluded.

**Fig. 1:**
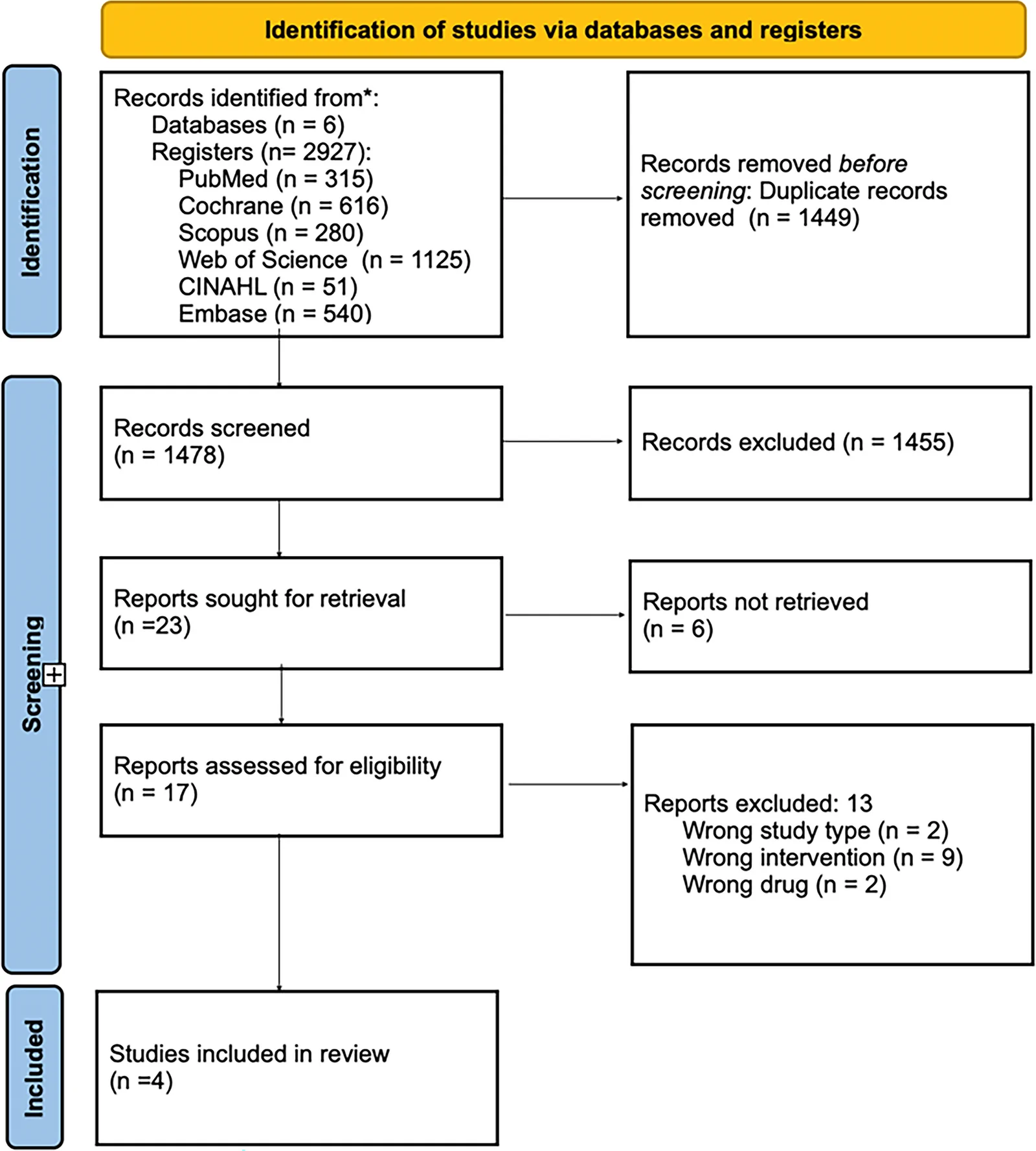
PRISMA 2020 Flow Diagram of Study Selection. Flow diagram illustrating the study selection process for the systematic review. A total of 2927 records were identified through searches of seven databases. After removing 1449 duplicates, 1,478 records were screened by title and abstract. Of these, 23 full-text articles were sought, but* 6* could not be retrieved. Among the 17 assessed for eligibility, 13 were excluded based on predefined criteria (intervention: *n* = 9; comparator: *n* = 2; study design: *n* = 2). In total, 4 studies were included in the systematic review. This process followed PRISMA 2020 guidelines

We sought retrieval of 23 full-text reports; however, 6 could not be retrieved. The remaining 17 reports were assessed for eligibility. Of these, 13 were excluded for the following reasons: wrong study type (n = 2), wrong intervention (n = 9), and wrong drug (n = 2). Ultimately, 4 studies met the inclusion criteria and were included in the review (Fig. 1).

### Included studies

A total of 288 children, aged 1–17 years, were included across four studies comparing once-daily versus divided-dose prednisolone regimens (Table 1). In all studies, the total daily dose was equivalent (2 mg/kg/day or 60 mg/m^2^/day), with differences lying in how the dose was administered.

**Table 1:** General outcomes

Author, Year	Country/Setting	Study design	Population (N)	Age range (Y.)	Single dose***	Divided dose***	Follow-up	Time to remission	Relapse outcomes	HPA suppression/AEs
Ekka et al. (1997) [18]	India/relapses	RCT	N: 47 Single: 23 Divided: 24	1–17	2 mg/kg/d (morning) for 2 w. followed by 1.5 mg/kg on alternate days for 4 w	2 mg/kg/d (three doses)	9 m	Single: 8.6 ± 2.8 d vs. Divided: 8.5 ± 2.9 d (p = 0.94, study power 96%)	N (relapse) Single:18 Divided: 20 Frequent relapses (≥ 3): 7 in the single-dose group vs. 8 in the divided-dose group Relapse rate: Single:1.7 Divided: 1.9 over 9 m (p = 0.85)	Serious infections were present in 2 patients of each group Obesity: Single: 2 Divided: 5 No significant differences
Sanousy et al. (2015) [8]	Egypt/initial or infrequently relapse	Prospective, comparative, non-randomized study	N: 30 Single: 15 Divide: 15	1–8	60 mg/m^2^/d max 80 mg (morning) for 4–6 w Followed by 40 mg/m^2^/d for 4 w	60 mg/m^2^/d max 80 mg (morning) for 4–6 w Followed by 40 mg/m^2^/d for 4 w	Until remission	Single: 3.2 ± 0.77 w Divided: 3.3 ± 1.35 w p = 0.77	NR	HPA axis suppression was not assessed AEs: No steroid toxicity Impetigo: Single:0 Divided:1
Weerasooriya (2023) [19]	Sri Lanka/relapses	RCT, single center	N: 96 Single: 55 Divided:49 104* episodes	1–14	60 mg/m^2^(morning) until remission**	60 mg/m^2^ divided in 2/3 AM + 1/3 PM until remission**	NR	Single: 9.74 ± 3.72 d Divided: 8.02 ± 1.58 d (p = 0.001) Divided significantly shortened time to remission compared to single morning	No relevant difference during follow-up	HPA axis suppression was not assessed AEs: No significant differences in safety profile between groups
Khan (2023) [6]	Kolkata India/first episode	Open label RCT	N: 60 Single: 30 Divided: 30	1–12	2 mg/kg/d (morning) for 6 w., followed by 1.5 mg/kg on alternate days for 6 w	2 mg/kg/d two doses for 6 w. followed by 1.5 mg/kg/d for 6 w	NR	Time to remission was similar in both groups	Median time to first relapse Single:131 d Divided: 131 d p = 0.002	Greater HPA suppression in divided vs. single (p = 0.02). Other AEs – Infections, Cushingoid features, behavioral changes, and GI symptoms – occurred at comparable rates.

^*^Some patients experienced more than one relapse; total relapse episodes = 104

^**^After remission, all patients were shifted to alternate-day prednisolone 60 mg/m^2^ as a single morning dose (Weerasooriya, 2023)

^***^All doses were Prednisone

*NR* Not Reported, *AE* Adverse Events, *Y* Years, *W* weeks, *D* Days

### First-episode disease

Khan et al. randomized 60 children with their first episode of NS to receive either a single morning dose (60 mg/m^2^/day once daily) or the same dose split into two equal morning and evening doses. Treatment was given for 6 weeks, followed by 6 weeks of alternate-day dosing. All children achieved remission, and overall relapse rates were similar. However, among children who relapsed within 6 months, the time to first relapse was significantly shorter in the divided-dose group (28 vs. 131 days, p = 0.002) [6].

Sanousy et al. prospectively studied 30 children with either first or infrequently relapsing disease, comparing a single morning dose (60 mg/m^2^/day) with the same total dose divided into three equal daily doses. No differences in time to remission were observed, and no steroid toxicity was reported. One case of impetigo occurred in the divided-dose group [8].

### Management of relapse episodes

Trials that have compared different steroid schedules during relapse treatment show mixed results. In an early study, Ekka et al. found no difference between once-daily and thrice-daily dosing: both groups achieved remission in just over 8 days, and short-term outcomes were similar [18]. More recently, Weerasooriya et al. reported a modest but significant benefit with divided dosing, with remission occurring nearly two days earlier on average (8.0 vs. 9.7 days, p = 0.001) [19]. The advantage was most evident in children aged 1–5 and 10–14 years. Importantly, this faster response was not associated with an increase in adverse events.

### Relapse recurrence and long-term outcomes

Whether the dosing strategy during relapse treatment affects the risk of future relapses is less clear. Ekka et al. followed children for nine months and found no differences in relapse frequency or cumulative steroid exposure between regimens [18]. Sanousy et al. [8] did not provide follow-up data, and relapse frequency was not a focus of the Weerasooriya trial [19]. In a separate study of first-episode disease, Khan et al. reported overall relapse rates were similar between single and split dosing, but among children who relapsed within six months, those treated with divided doses relapsed significantly sooner (median 28 vs. 131 days, p = 0.002) [6].

### HPA axis suppression and adverse events

Across studies, adverse event rates were generally low. Sanousy et al. reported no steroid-related toxicity, with one impetigo case in the divided-dose group [8]. Ekka et al. [18] and Weerasooriya et al. [19] both found comparable adverse-event profiles between regimens. In contrast, Khan et al. [6] demonstrated significantly greater HPA axis suppression at 6 weeks with divided dosing compared with a single morning dose (100% vs. 83%, p = 0.02).

### Risk of bias

**Fig. 2:**
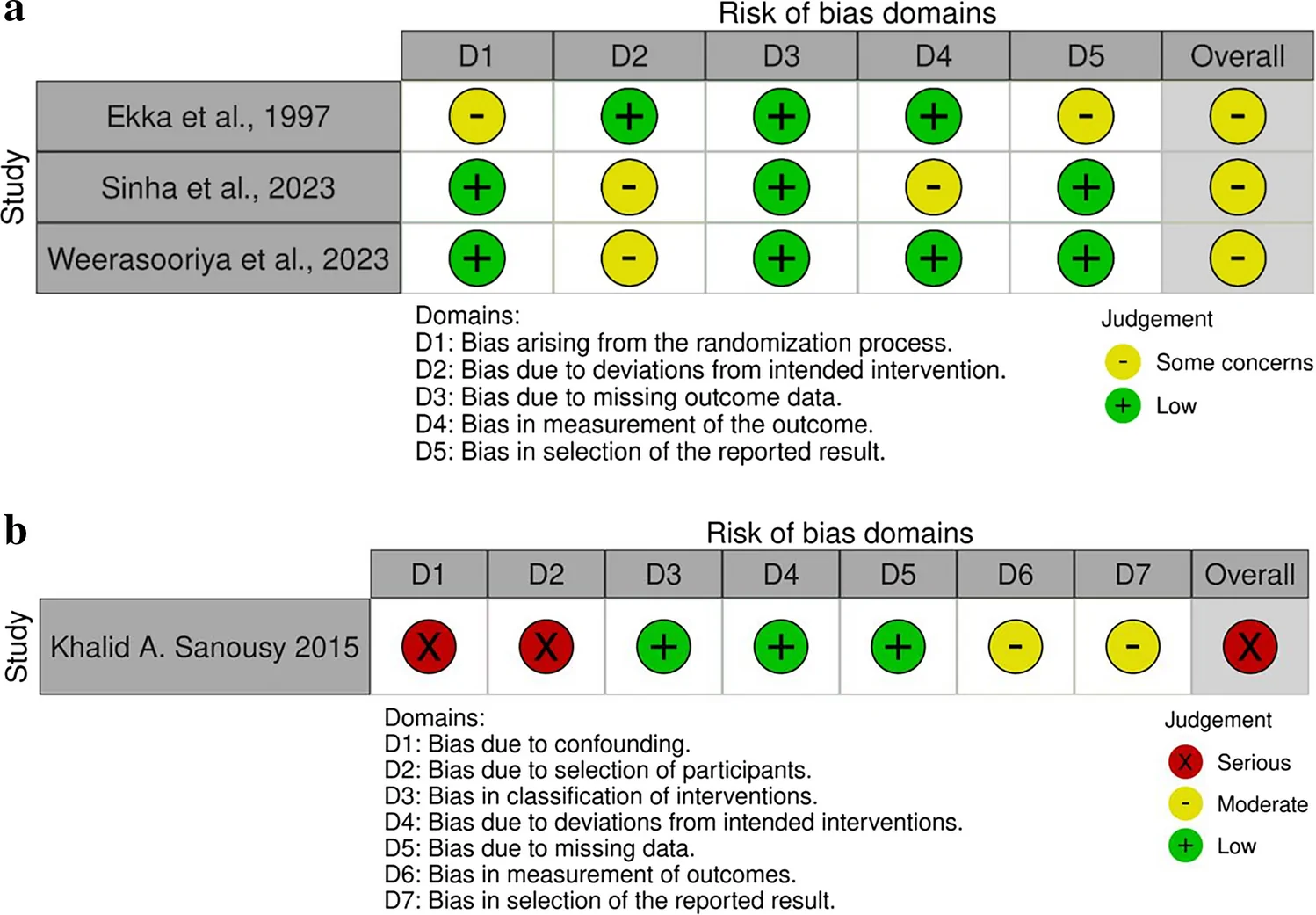
**a.** Risk of bias assessment for randomized controlled studies using the RoB 2.0 tool. Risk of bias across seven Rob 2.0 domains: D1 (confounding), D2 (participant selection), D3 (intervention classification), D4 (deviation from intended intervention), D5 (missing data), D6 (outcome measurement), D7 (reported result selection). Judgment was classified as Low (+), Moderate (-), or Serious (X). Overall risk of bias was based on the most severe judgment across domains. **b**. Risk of bias assessment for randomized controlled studies using ROBINS-I. Risk of bias was evaluated across five domains: D1 = bias arising from the randomization process (or confounding in non-randomized studies); D2 = bias due to deviations from intended interventions; D3 = bias due to missing outcome data; D4 = bias in measurement of outcomes; and D5 = bias in selection of the reported result

The three randomized trials (Ekka [18]; Weerasooriya [19]; and Khan [6]) were judged to have “some concerns,” primarily due to their open-label design and lack of blinding. The non-randomized prospective study (Sanousy [8]) was assessed at high risk of bias due to absence of randomization, small sample size, and incomplete outcome reporting (Fig. 2a and 2b).

## Meta-analysis

### Time for remission

**Fig. 3:**
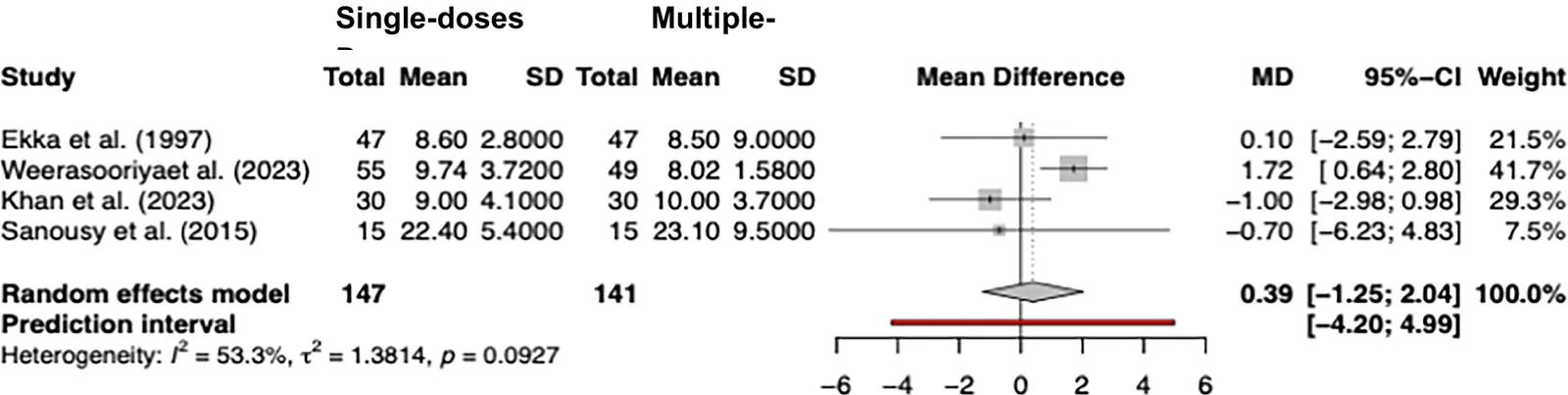
Forest Plot. Forest plot comparing once-daily (experimental) versus divided-dose (control) prednisolone regimens in children with steroid-sensitive nephrotic syndrome. Each square represents the mean difference (95% CI) in time to remission between the two dosing strategies, with the size of the square proportional to study weight. The diamond represents the pooled mean difference under a random-effects model. The vertical red line indicates no difference (mean difference = 0). *Abbreviations:* MD = Mean Difference; CI = Confidence Interval; SD = Standard Deviation

**Fig. 4:**
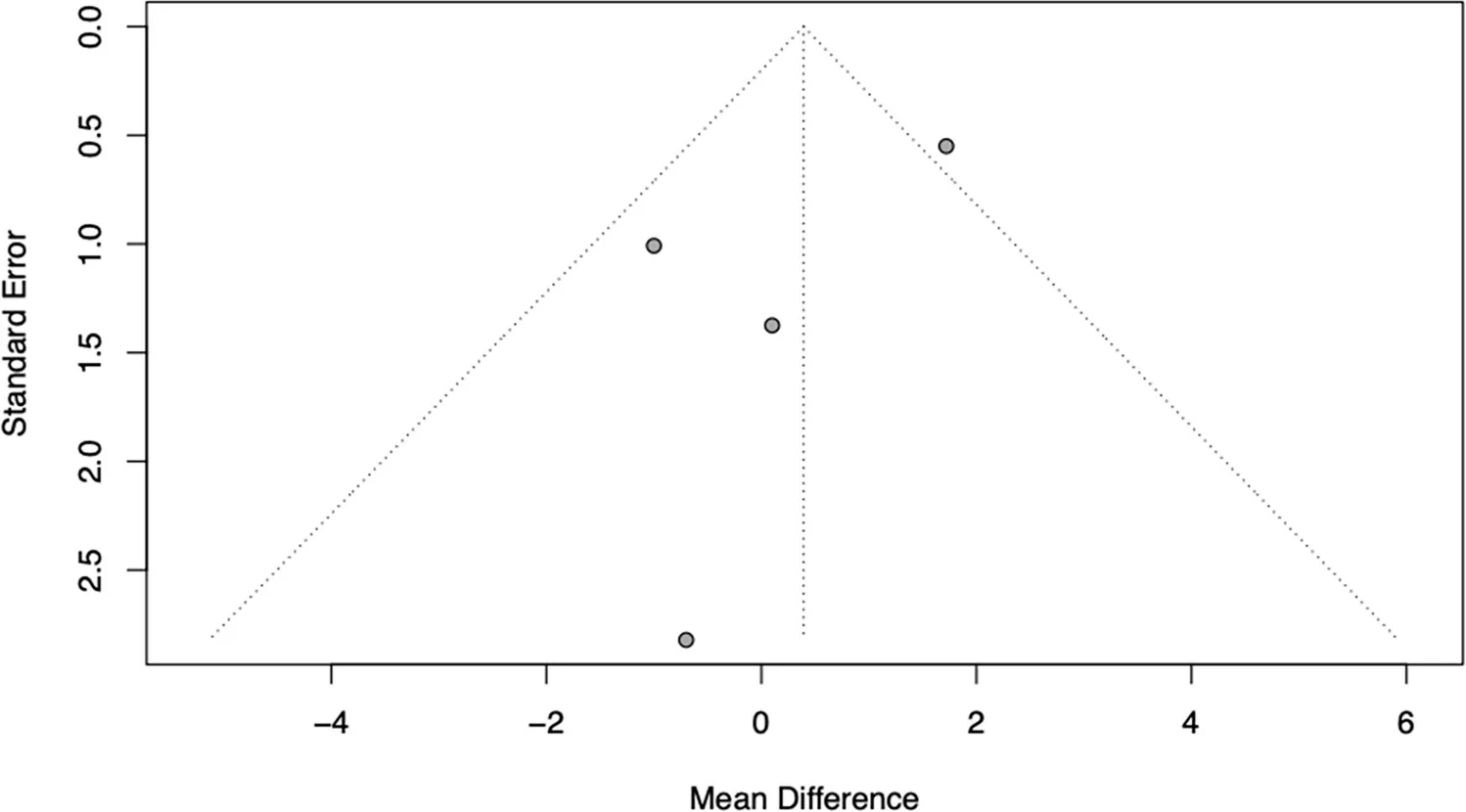
Funnel plot. Funnel plot illustrates distribution of studies, with mean difference on the x-axis and standard error on the y-axis. The plot helps assess potential publication bias and heterogeneity among the included studies

We conducted a meta-analysis comparing single versus divided doses of prednisone in the treatment of NS. Four studies were included, comprising a total of 288 participants (147 in the single-dose group and 141 in the divided-dose group). The pooled mean difference (MD) in time to remission was 0.39 days (95% CI: −1.24 to 2.03; p = 0.63, I2 = 53.3%; Fig. 3). Visual inspection of the funnel plot suggested asymmetry among the included studies (Fig. 4), indicating potential publication bias. Due to the small number of studies (n = 4), Egger’s test for small-study effects could not be performed. Subgroup analyses were also not conducted due to the limited number of studies. Sensitivity analysis revealed that two studies, Weerasooriya et al. [19] and Khan et al. [6], had a significant influence on the overall findings and heterogeneity estimates; excluding these articles was not feasible due to the low number of studies remaining (Supplementary Table 1).

## Discussion

Current international guidelines, including the IPNA 2022/2023 clinical practice recommendations and the KDIGO 2025 guideline, support once-daily morning prednisolone as standard practice for both first episodes and relapse management of SSNS, while acknowledging that divided dosing has also been used in specific clinical contexts [20, 21]. This approach reflects a balance between physiology and practicality: morning dosing aligns with the natural cortisol surge, may reduce the risk of HPA axis suppression, and is simpler for families to follow consistently. Notably, KDIGO’s most recent evidence tables explicitly compare once-daily and divided regimens, underscoring the clinical relevance of this question [20, 21].

Our findings support the physiological rationale for once-daily morning dosing. Endogenous cortisol secretion peaks early in the morning and administering prednisolone at this time synchronizes exogenous exposure with circadian rhythms, potentially reducing HPA axis suppression. This pattern was demonstrated in the only trial that objectively assessed adrenal function, where divided dosing led to significantly greater suppression than single-dose regimens [6]. These results strengthen the biologic justification for morning dosing as the safer strategy for endocrine preservation. In contrast, divided schedules maintain glucocorticoid receptor occupancy throughout the day and were associated in some relapse-focused trials with modestly faster remission [18, 19], though this benefit must be weighed against higher rates of HPA-axis suppression and the absence of differences in long-term outcomes [6].

Efficacy outcomes showed more variability across studies. In first-episode disease, time to remission was similar between once-daily and divided regimens, suggesting that increasing within-day dosing frequency does not accelerate initial disease control [6, 8. In relapse settings, two trials noted a one- to two-day faster remission with divided dosing, a difference that may reflect prolonged receptor engagement [18, 19]. However, its clinical significance remains uncertain given the lack of differences in cumulative steroid exposure or longer-term relapse patterns.

Relapse outcomes further highlight that any short-term benefit of divided dosing must be interpreted with caution. In first-episode disease, children receiving once-daily dosing experienced a substantially longer interval to first relapse among those who relapsed [6] suggesting that physiological dosing schedules may confer a protective effect. Relapse-focused trials found no significant differences in long-term recurrence, and one study did not report relapse follow-up [8]. Taken together, relapse dynamics do not consistently favor divided dosing and in some cases may support single-dose regimens [22, 23]. Safety findings across the included studies further reinforce once-daily morning prednisolone as the preferred default approach. Adverse events were generally mild and similar between groups [8, 18, 19]. However, the markedly higher rate of HPA-axis suppression associated with divided dosing​ underscores the importance of aligning steroid administration with circadian biology, especially for children who are particularly vulnerable to long-term endocrine effects [6].

Despite these meaningful insights, the evidence base remains limited. The included studies are few, often single-center, open-label, and heterogeneous. This heterogeneity also reflects the inherent clinical differences between first episode and relapse episodes. Although both contexts evaluate remission under equivalent steroid doses, their underlying biology and clinical trajectories may differ, and this limitation is now explicitly acknowledged. Our meta-analysis results were sensitive to the largest trials, reflecting the vulnerability of pooled estimates when the available literature is sparse. These limitations reinforce the need for adequately powered multicenter randomized controlled trials with standardized dosing schedules, extended follow-up, and inclusion of patient-centered outcomes such as adherence, caregiver burden, and quality of life. Importantly, ongoing studies, including the RESTERN trial in the Netherlands and new work in India, are evaluating prednisolone regimens that vary by both duration and schedule. Their results may provide more definitive evidence that is currently lacking [24, 25].

In summary, the currently available evidence suggests that once-daily morning prednisolone may offer practical and physiological advantages, particularly with respect to aligning treatment with circadian cortisol rhythms. However, the existing trials are few, heterogeneous, and generally underpowered, making it difficult to establish a preferred dosing regimen with confidence. Although divided dosing may shorten time to remission by one to two days in some relapse episodes, this finding is inconsistent, and its clinical relevance remains uncertain. Evidence regarding HPA-axis suppression is also extremely limited, with only one study providing an objective assessment. Given these uncertainties, larger, blinded, and methodologically rigorous randomized trials are needed to determine whether potential differences in remission speed or endocrine effects translate into meaningful improvements in outcomes such as duration of illness, school attendance, treatment burden, and long-term adrenal health.

## Conclusion

This systematic review indicates that single-dose and split-dose prednisolone regimens achieve comparable remission rates in children with nephrotic syndrome. The choice of schedule may therefore depend more on safety and practicality than on efficacy. A single morning dose may improve adherence and better preserve adrenal function, particularly in first-episode disease. In contrast, split dosing may be considered in relapse settings where shortening time to remission is a clinical priority, although the clinical significance of a one- to two-day difference remains uncertain. The marked heterogeneity of current protocols, small single-center studies, and short follow-up periods limit the strength of existing evidence. Future research should focus on large multicenter randomized controlled trials with standardized dosing strategies, longer follow-up, and inclusion of patient-reported outcomes to provide definitive guidance for clinical practice.

## Limitations and practical implications

Only four studies met the inclusion criteria, limiting the available data and preventing robust subgroup or sensitivity analyses ​[6, 8, 18, 19]​. The small, single-center nature of the trials, heterogeneous dosing schedules, and short follow-up periods (typically 6 to 9 months) reduce the strength of the evidence. Importantly, only one trial objectively assessed adrenal function, and none reported patient-centered outcomes such as adherence, caregiver burden, or quality of life. Our meta-analysis also showed that the pooled effect is highly sensitive to the inclusion of the largest 2023 study; when this trial is excluded, the results approach statistical significance, which underscores how risk of bias becomes critical when the evidence base is so limited. Despite these limitations, the absence of prior meta-analyses on this topic underscores the novelty and relevance of this work, which provides the first synthesis of available evidence and highlights key gaps for future multicenter RCTs with standardized protocols and longer follow-up. Collectively, the evidence suggests that remission rates are largely equivalent, with differences emerging mainly in time to remission, which was faster with divided dosing in one relapse trial, and in endocrine safety, with less HPA suppression observed in first-episode cases treated with single morning dosing.

## Supplementary Information

Below is the link to the electronic supplementary material.

Graphical abstract (PPTX 177 KB)

Supplementary file2 (DOCX 15 KB)

## Data Availability

All the data used to perform this analysis are publicly available in the referenced articles. The rest of the material is at the author's disposition upon reasonable request.

## Abbreviations

CINAHL: Cumulative Index to Nursing and Allied Health Literature
g/dl: Grams per deciliter
HPA: Hypothalamic-pituitary-adrenal
IPNA: International Pediatric Nephrology Association
KDIGO: Kidney Disease: Improving Global Outcomes
mg/kg: Milligrams per kilogram
mg/kg/day: Milligrams per kilogram per day
mg/m^2^: Milligrams per square meter
mg/mmol: Milligrams per millimole
NS: Nephrotic syndrome
PRISMA: Preferred Reporting Items for Systematic Reviews and Meta-analyses
PROSPERO: International Prospective Register of Systematic Review
RCTs: Randomized Clinical Trials
RoB: Risk of Bias
SSNS: Steroid-sensitive nephrotic syndrome
UPcr: Urine protein to creatinine ratio
vs: Versus
NR: Not reported
