# Supplementary material for: Single-dose versus multi-dose steroid therapy in pediatric nephrotic syndrome: a systematic review and meta-analysis
Source: Pediatr Nephrol. 2026 Feb 28;41(11):3841–51. doi: 10.1007/s00467-026-07175-z (PMC13612631; doi:10.1007/s00467-026-07175-z)
Supplement: Supplementary file 1 [file 467_2026_7175_MOESM2_ESM.docx]

**Supplementary Material**

**Supplementary Table 1.** Sensitivity analysis

| **Author** | **Effect (95% CI: Lower; Upper)** | **I²** | **DFFITS** | **Cook's D** | **QE (del)** | **Is Influential** |
| --- | --- | --- | --- | --- | --- | --- |
| Ekka et al. (1997)  [18] | 0.3793 (-1.8037; 2.5622) | 66.7 | 0.0151 | 0.0003 | 6.0019 | No |
| Weerasooriya et al. (2023)  [19] | -0.6218 (-2.1531; 0.9095) | 0.0 | 2.8576 | 1.4672 | 0.4171 | Yes |
| Khan et al. (2023)  [6] | 1.2485 (-0.0813; 2.5783) | 0.0 | -1.3652 | 1.0408 | 1.7837 | Yes |
| Sanousy et al. (2015)  [8] | 0.4636 (-1.3186; 2.2458) | 67.1 | -0.0829 | 0.0070 | 6.0755 | No |

A sensitivity analysis was performed to assess the impact of removing individual studies on the overall pooled estimate. Influence diagnostics were applied to identify studies that contributed to heterogeneity. The “Is Influential” indicator denotes whether a study substantially altered the results.
